# Fake leadership influence on organizational destruction in Higher Education Institutions (HEIs)

**DOI:** 10.1371/journal.pone.0321194

**Published:** 2025-04-23

**Authors:** Agnieszka Bieńkowska, Katarzyna Tworek

**Affiliations:** 1 Faculty of Management, Wrocław University of Science and Technology, Wrocław, Poland; 2 Faculty of Management, Wrocław University of Science and Technology, Wrocław, Poland.; Federal University of Santa Maria: Universidade Federal de Santa Maria, BRAZIL

## Abstract

The role of leadership in Higher Education Institutions (HEIs) is pivotal. While much research emphasizes the beneficial effects of leadership, the negative impacts of destructive leadership styles remain less explored. This paper investigates the concept of fake leadership - a form of destructive leadership characterized by leaders’ intent to engage in harmful behaviors while maintaining a facade of authenticity - and its influence on organizational destruction in HEIs. Drawing on the Toxic Triangle Framework, this study examines how fake leadership undermines intra-organizational trust and job performance, ultimately fostering systemic inefficiencies and organizational decline. The two-step empirical study was conducted with 529 employees from HEIs across Europe (France, Poland, Spain, and the United Kingdom) to verify the hypotheses. Statistical reasoning was based on linear regression analysis with mediators. The results confirmed that fake leadership significantly and positively influences organizational destruction. This effect is mediated by a decline in intra-organizational trust and job performance. This study contributes to the literature by introducing fake leadership as a distinct destructive leadership style in HEIs, providing a tailored framework for understanding its role in enabling organizational destruction, and underscoring the critical role of diminished intra-organizational trust and job performance in deepening these adverse effects. Practical implications emphasize the need for ethical leadership, rigorous selection processes, and proactive measures to safeguard institutional integrity and resilience.

## Introduction

Much has been written about leadership in organizations. Higher Education Institutions (HEIs), like any organization, require a conscious approach to leadership [1]. It means that the influence of leaders on followers with the goal of moving together in the direction the HEI has chosen as strategic. Specific leadership styles have been identified as appropriate for HEIs, combining the contemporary distinctive features of authentic [2, 3] and distributed [4] leadership emphasizing, in particular, a high degree of autonomy, as well as a sense of responsibility at all levels of leadership. This leadership concerns both the hierarchical and administrative relationships at the HEI and the diffuse master-apprentice relationships, as well as the special trust-based ties that exist within the academic community. In the literature, this style is often referred to as academic leadership [1, 5]. It is a response to the contingency theory [6] applied in organizations, according to which the solutions implemented in an organization must correspond to its specifics. HEIs are, without any doubt, specific organizations [1]. However, that is not only because of a specific type of activities carried out in HEIs, but first and foremost because of the type of resources that are at their disposal and enable them to function. Employees are the key resources in HEI, especially research and teaching staff (professors, assistant professors, assistants), who are characterized by unique competencies that are difficult to replace on the labor market, and which determine the achievement of the goals set by the university [7]. Leadership, defined as academic leadership, makes it possible to influence HEIs employees in such a way that, by influencing their job-related attitudes, will contribute to an increase in job performance, and organizational performance in all areas of the HEIs’ activities.

The problem arises when, whether intentionally or not, the leader uses a style far different from the style recommended and widely recognized as effective, especially when it is a style bearing the hallmarks of a destructive leadership style – as a rule, negatively affecting the organization. There can be many reasons for the use of destructive leadership styles in HEIs. The literature points to high expectations, limited resources, and long-term academic competition [8], pressures and new tensions that are constantly arising [8, 9], positional games of employees [10] and the personality of the leader [11]. However, regardless of the reasons, destructive leadership styles introduce various problems into the organizations, connected with leaders’ traits, as well as actions towards employees and the organization [12]. They do not only have an adverse effect on organizational performance, as well as other outcome parameters relating to the organization as a whole, but due to their negative influence, they can point the organization into the road to decline, causing its destruction (e.g., [13, 14]). One of the most destructive leadership styles for HEIs is fake leadership [12], which is characterized not only by the intent of the leader to engage in negative behaviour towards employees and organization, but with behaviours aimed at hiding such intent – and it will be a subject of this study.

There is still a limited number of research on the relation between leadership styles and HEI performance available in the contemporary literature. Most of them indicate that various well-suited leadership styles positively influence HEIs performance through various mediator, including work motivation [15], job satisfaction [16], social innovation [17]. Almost all of them relate to the positive influence of leadership styles on HEI performance (including the performance of HEI employees). The notion of negative influence of leadership styles on HEIs is much more rarely addressed in the literature, especially in case of destructive leadership styles. Previously it was most commonly connected to threat to leader’s power [18], conflict [19] or students’ perspective (e.g., [20]). It starts to get some broader attention (e.g., [21, 22]), however the conclusions remain fragmentary and usually relate to narrow context. Various existing studies still do not allow for the systemic analysis of the issue, especially since they lack the perspective of leader intent (central for the proper understating of destructive leadership). Moreover, they also concern only diminished performance, hardly ever address the issue of destructive leadership styles as a source of potential destruction within HEIs, which remain especially susceptible according to Toxic Triangle Framework [23]. It shows a clear and vast research gap, concerning lack of research on the dark side of leadership in HEIs and its negative influence on HEI employees and organization as a whole, causing organizational destruction. **Therefore, the aim of the paper will be to examine the role of fake leadership in creating organizational destruction of HEIs.** Such aim will be fulfilled with critical literature review used as a basis of hypotheses development concerning the role of fake leadership in fostering organizational destruction, and empirical study implemented among over 500 employees of HEIs from European countries aimed at their verification.

## 1. Theoretical background

### 1.1. Higher Education Institutions (HEIs)

HEIs are organizations that, on the one hand, can be described by traditional models of organization (such as the Leavitt model), while on the other, can be described taking into account its specifics, which clearly distinguish it from other organizations, especially when referring to the analysis of the functioning of the academy. The two most important missions of HEI are teaching and research [24]. Highly qualified staff, academic ethos, autonomy in action, democratization of decisions, pursuit of truth and teaching of truth (as an object of activity), academic community, or, finally, the academic culture - are a set of artifacts that are in vain to be found in economic entities or other organizations functioning today. Moreover, it is impossible to reduce a modern HEI to the role of a corporation - this is contradicted primarily by the universal values that are still the pillars of HEI. The canon of fundamental values, which “determines the peculiarity of the university, its considerable durability and resistance to the impact of destructive factors, as well as its indispensability for the duration and development of society (...) a canon that was developed by many generations of those who co-created the university, and fidelity to these values determined the university’s ‘long history of duration’” [25]. In this context, a modern HEI should be understood as “drawing on tradition, an autonomous and socially responsible organization; based on supranational, universal values such as truth, morality, honesty, respect; sensitive to the well-being of each member of the academic community both individually and collectively; realizing teaching and research work on a complementary basis and meeting in a balanced way the current and future needs and expectations of all stakeholders” [26] and thus participating in shaping the reality around it.

As an organization, a HEI requires a professional approach behind management. However, it should take into account the specifics and internal and external conditions of the academy. Increasingly, management theory and practice emphasize the need to incorporate strategic management tools, personnel management tools (including personnel selection, shaping career paths, or motivating employees), as well as financial management or infrastructure management (including IT), not to mention the need to include marketing management tools or shaping quality assurance systems [27, 28] in the practice of higher education. Therefore, it is not surprising that discussion of the functioning of a modern HEI cannot omit leadership as a process of influencing employees.

#### 1.1.1. Leadership in HEIs.

The leadership in HEIs has been the focus of scholarly interest over the last decades (e.g., [16, 29–33]). Leaders play a crucial role in contemporary HEI [30, 33]. Proper leadership fosters a positive culture in higher education institutions (HEIs) that encourages collaboration, a sense of belonging, commitment, and an environment where scholarly pursuits thrive and collective achievements are celebrated [34]. The positive influence of leadership styles on the performance of entire organization was proven to be especially true for HEIs [35]. That is mainly because leaders are responsible not only for decision-making while considering the interests of stakeholders and the organization [36], but also (or above else) for establishing strategic action plans, articulating visions, missions, and values [30, 33]. In addition to indicating the importance of leadership for HEIs, the current discussion in the literature highlights that different positive and well-suited leadership styles have varying influence on HEI performance [37] through various factors (including work motivation [15], job satisfaction [16] and other factors) and calls for a deeper exploration of these effects [29, 33]. Especially the guidelines of New Public Management (NPM) underline the need to identify the leadership style most suitable for HEIs, which enabled the researchers’ debate [20, 38]. Saad [38] indicate that strategic leadership is the most appropriate leadership style for HEI, underlining the need for strategic management in the context of future-oriented approach toward a development, and the use of intellectual capital and creativity. Rehman and Iqbal [39] indicate that leadership oriented toward knowledge is the most appropriate leadership style for HEI, showing its positive influence on organizational performance.). Angelo & McCarthy [40] indicate that shared leadership is the most appropriate leadership style for HEI, also showing its influence on improving the performance of virtual teams. Various studies indicate other leadership styles as the most appropriate leadership style for HEI and offer the analysis of its influence on HEIs and verification of its positive influence on HEIs performance, i.e., transactional leadership (e.g., [32]), charismatic leadership [41], collaborative leadership [42], sustainable leadership [43] and distributed leadership [22, 44]. There is even a tendency to shape a specific leadership style suitable for HEIs - academic leadership [5].

#### 1.1.2. Fake leadership in HEI.

However, leadership is a notion connected not only to positive traits and behaviors of a leader, but also to the negative ones [14, 45]. Destructive leadership styles are defined by Einarsen [46] as “the systematic and repeated behavior by a leader, supervisor, or manager that violates the legitimate interests of the organization by undermining and/or sabotaging the organization’s goals, tasks, resources, and effectiveness, and/or the motivation, well-being, or job satisfaction of subordinates”. However, this field of study still lacks a complex systemic approach and various specific leadership styles emerge within the group of destructive leadership styles [45].

The notion of destructive leadership styles in HEIs and its influence on those institutions are also getting some scholarly interest, especially during recent decade, however it still remains much less discussed than the positive aspects of leadership in HEIs, despite the growing need for that [21, 22, 47]. Dopson et al. [48] point out that “HEI settings change radically throughout the world, HEI professionals are operating in more uncertain environments, and leaders are taking increasingly complex and diverse approaches to their leadership roles.”, which makes them more prone to various negative behaviors. Recent studies discuss the range of factors contributing to the emergence of various destructive leadership styles in HEIs. Among these, Smith and Fredricks-Lowman [19] discuss specific organizational culture prone to conflict and toxic leaders’ behaviors. Ryan et al. [18] highlight the perception of a threat posed by subordinates who are believed to be more competent or capable.

Various studies concerning the destructive leadership styles in HEIs, characterized by harmful and counterproductive behaviors, indicate mechanisms of its negative influence on employees and organization (e.g., [18, 21, 22, 49]). However, even though various approaches emerged, the existing research does not clearly discuss the issue of intent of the destructive leaders in HEIs. The notion of intent is a characteristic of leadership style underlined by various meta-analysis made in that field of study [45, 50]. However, almost none of them distinguishes between the intent to engage in negative behaviors and intent to hide that fact and most of the do not analyze the intent as one of the characteristics of leadership. It remains a reason for lack of consistency of this field of study and constitutes an important issue, as negative and hidden intent is one of the key sources of organizational pathologies [51]. Various research emerged during the recent decade on sources and effects of various pathologies in education sector (including HEIs), showing their negative effects on HEIs (e.g., [52–54]), confirming that. Hence, it is important to properly address the issue of intent in the context of destructive leadership styles influence on HEIs.

Therefore, this paper concerns the analysis of the specific destructive leadership style in HEIs – fake leadership. It is assumed that **fake leadership is characterized not only by the intent of the leader to engage in negative behaviour towards employees and organization, but with behaviours aimed at hiding such intent** and appearing to be an authentic leader, engaging in positive behaviours towards employees and organization [12]. Therefore, there are three characteristics of **fake leadership**, which distinguish that concept from those already appearing in the literature:

1. The existence of a **full spectrum of traits and behaviours** (not just some isolated ones), which are characterized by the focus on individual goals of the leader (regardless of their importance for the organization or employees) coupled with anti-employees and anti-organizational behaviours. The **fake leader** is characterized by negative traits that make the leader more susceptible and prone to negative behavior toward employees and the organization (lack of empathy, self-involvement, high but fragile self-esteem), intention to win combined with a low sense of responsibility, excessive sensitivity to criticism and need for approval, and insincerity and ability to pretend. The negative **behaviors directed against employees** concern creating and maintaining fear among employees, humiliating employees, gathering information to use against employees, demeaning, marginalizing or degrading employees, exploiting employees, destroying labor relations, and most importantly: misleading and gassing employees. The negative **behaviors against the organization** concern manipulating information, lack of communication and transparency, volatility and inconsistency, and destroying communities [12].
2. The **intent** of the leader **to engage** in negative behaviours towards employees and organization [12].
3. The mindfulness of the leader **to hide that intent** from employees and appear to be an authentic leader engaging in positive behaviours towards employees and organization [12].

### 1.2. Fake leadership and organizational destruction

#### 1.2.1. Organizational destruction.

Classical overall organizational outcome parameters include measuring its performance in the context of its goals [55], performance relating to the activities it undertakes [56], the synthetic parameter organizational performance, or organizational reliability, which should be assessed as particularly important in crisis situations causing disruption to the organization [57]. All of them assume, at base, the measurement of the performance of the organization on a development path (regardless of the stage of the life cycle [58]. However, this is not always the case. Sometimes ineffectiveness occurs [59]. What is missing, however, is an outcome parameter that shows an organization that does not so much fail to achieve organizational performance, or reliability, but records the negative outcomes of its operations on the path of decline, not development. It should be similar to dissatisfaction [60], demotivation [61], or other negative job-related attitudes are described in the literature with regard to employees.

Organizational destruction may be such parameter concerning the organization, showing the level of deteriorations of the organization on the path to decline. Organizational destruction assumes that, as a result of intentional or unintentional actions of the management, the organization is exhibiting characteristics that prone it to be on the path to decline. Those characteristics should be analyzed precisely in four areas of the organization according to Leavitt’s concept: goals, people (including management), structure and technology [62], as decline concern all area of organization.

HEIs are especially vulnerable to the negative influence of characteristics that enable the organizational destruction, as they are organizations highly exposed to positional games of employees [10]. Moreover, they are usually employing people with high drive for excellence and above-average self-confidence and ego [11], not necessarily led by people with management competences, but by scientists. Therefore, when power and leadership is placed in the wrong hands, it may trigger the destruction of organization [11] and enable those characteristics of organizational destruction to appear. Therefore, there is a need to identify those characteristics specific for HEIs, as those organizations remains very specific also in such context [63].

Thus, divested according to Leavitt’s concept, the construct of organizational destruction for HEIs will imply the following characteristics:

1. (synthetic approach) The perceived and identified perception of the actions taken by the management as destructive actions.
2. (goals) The perceived discrepancy between the organization’s stated/planned goals and the actions taken (intentionally or not) by management.
3. (people) The perceived lack of management competence and increased level of willingness to leave among employees (however, due to specificity of HEIs, it does not necessarily translate into their actual departure).
4. (structure) Lack of adherence to organizational rules derived from the organizational structure.
5. (technology) Loss/degradation of organizational resources, especially technological resources.

#### 1.2.2. The influence of fake leadership on organizational destruction in HEIs.

“Traditionally, leadership research focused on the analysis of leader traits, behaviours, and situations that contribute to individual and organizational effectiveness” [59]. Fake leadership changes the rules of the game in an organization [12]. This is because it is inherently unclear what the organization’s goals are, as they are intentionally concealed by the fake leader. Therefore, fake leader pursues goals known only to herself/ himself. It is known, however, that these are goals that will, as a rule, provoke opposition from the community – especially concious in HEIs [10] – so there is a need to hide the true intentions of the fake leader. Such situation may lead to organizational destruction, because employees are able - despite the lack of full understanding of the real plans of the fake leader - to notice some negative behaviors of the fake leader both towards them and the organization. This, in turn, triggers not so much resistance as passivity and an intentional failure of employees to contribute to the fake leader’s true goals, with which they do not identify [12]. Therefore, organization is brought to a state of its destruction. The very specific variant of that situation occurs when the hidden goal of the fake leader is, in fact, to destroy the organization. In doing so, it is irrelevant what causes it: own motivations or pressuring external factors. Regardless of that, fake leader is intentionally working toward the deliberate destruction of the organization. Fake leadership here is not only a tool used to hide the true intentions. At the same time, gaslighting activities are carried out against the employees, which help create chaos and disbelief in their own perception of the situation. Employees become confused and lose confidence in the validity of actions previously judged to be right and unconsciously contribute to actions aimed at destroying organization.

Therefore, fake leadership itself significantly contributes to organizational destruction by fostering an environment of misaligned goals, incompetence, and systemic inefficiencies – elements crucial for organizational destruction. When the goals at various levels are not translated into real, actionable steps, it creates a disconnect between the institution’s strategic vision and its practical outcomes and fake leaders themselves further foster chaotic, random and destructive actions towards organization [12].

Destructive leadership behaviors consistent with fake leadership are particularly damaging in HEIs due to the unique social and organizational context in which leadership is exercised [18]. The structure of HEIs [64] and their inherently hierarchical nature [65] create environments where power dynamics and authority can become amplified. The way leadership is approached, the exercise of authority [66, 67], and the existing power relations between leaders and followers [18, 68] all contribute to the potential for harmful leadership practices. Additionally, employees of HEIs are especially vulnerable to specific situational factors within HEIs, such as high expectations, limited resources, and long-term academic competition, which can further facilitate the emergence of destructive leadership behaviors [8]. It is especially apparent since new pressures and new tensions are arising in the growing pace since the beginning of this century [8, 9]. This combination of institutional and interpersonal factors makes HEIs particularly susceptible to the negative effects of such behaviors, including organizational destruction [10]. Moreover, destructive leadership styles, including fake leadership, relate directly to Toxic Triangle Framework [23]. It illustrates the interplay between leaders, subordinates, and the organizational environment that may facilitate various destructive leadership styles. Padilla et al. [69] underlines that, “destructive organizational outcomes are not exclusively the result of destructive leaders but are also products of susceptible followers and conducive environments.” Hence, not only the environment of hierarchical organizations, such as HEIs, but its combination with certain leader traits (including their hidden intention central for fake leadership) and subordinate vulnerabilities, can in fact lead to organizational destruction [18]. Such view is confirmed by Schneider [70].

In view of all the above, fake leadership should be categorized as destructive leadership style. “Destructive leadership in this sense would be defined as behaviour that directly or indirectly prevents organizational (e.g., quality and quantity of work) and personal goal attainment of followers (e.g., job satisfaction)” [59]. Therefore, the following hypotheses can be formulated:

***H1a: The fake leadership is positively influencing organizational destruction in HEIs.***

#### 1.2.3. The influence of fake leadership on organizational destruction through intra-organizational trust in HEIs.

Intra-organizational trust can be defined as a multidimensional construct that reflects the willingness of individuals within an organization to be vulnerable to others, based on positive expectations about their intentions, behavior, and reliability [71]. It encompasses trust at various levels, including trust between employees, between employees and their leaders, and trust in organizational systems and processes. This trust is rooted in perceived competence, benevolence, integrity, and fairness, forming the foundation of professional relationships and organizational functioning [72, 73].

Tierney [74] stated that there is lack of conceptual and empirical research concerning trust in HEI and that statement still remains to be true, even though some studies emerged. However, it is usually connected with governance [75]. The literature on HEIs highlights the growing distrust among staff, linked to a shift away from academic collegiality toward market-oriented management strategies that prioritize corporate control and employment outcomes [76]. Authors are underlining the need to develop and implement leadership conducive for boosting intra-organizational trust [76, 77] in order to avoid organizational destruction. That is because the issue of intra-organizational trust is critical to the functioning of the HEIs [74]. In particular, the cooperation of teams developing new solutions or discovering scientific truths requires confidence in the proper attitudes and ethical actions of co-workers. This type of relationship requires trust between employees, often built up over years (which is allowed by the nature of the HEI as an organization). It is based on shared academic values, and not just on positive experiences or a system of legal restrictions saturated, in particular, with penalties for non-compliance with contracts and commitments made [78].

Hence, it allows to assume that destructive leadership styles, such as fake leadership, will most probably negatively influence intra-organizational trust. It is mainly due to various negative behaviors of fake leaders, especially those directly aiming at destruction of communities and mutual trust and ability and intent to deceive employees. It will strengthen the negative influence of such leadership on organization, increasing organizational destruction. Fake leaders’ essential action is aimed at breaking the bond between employees in the form of trust, which deepens distrust and mutual dislike between co-workers. Such actions precisely aimed at the loss of intra-organizational trust - if effective - lead to a spontaneous lack of communication between employees (additional, assuming that the fake leader also carries out actions directly hindering or preventing intra-organizational communication). As a result, it reflect on the sense of isolation of employees and prevent verification of their own views (being consciously misled by fake leaders) with those of co-workers [79]. Moreover, it seems especially important to underline that the negative intent, coupled with the intention to hide it – central for fake leadership – is especially harmful for intra-organizational trust [12], hindering their ability to maintain performance crucial for restraining organizational destruction. Moreover, behaviors typical for fake leadership create a culture of uncertainty, where decision-making appears erratic and unaligned with the formal structures and processes of the organization, enhancing lack of trust. Such lack of trust and randomness further destabilizes the HEI, leading to a breakdown in coherence and direction, increasing organizational destruction. It remains in line with the study of Maassen and Stensaker [80], who consider trust in governance of HEIs, underlining that without it, it is hard to establish the influence of leadership on organizational performance, and even further – it is easier to fall into organizational destruction due to lack of it.

Moreover, Bieńkowska and Tworek [12] proposed various models of fake leadership influence on job performance and organizational reliability, indicating the mechanism of such influence through various mediators, e.g., intra-organizational trust, job-related attitudes, anti-job-related attitudes, attitude towards errors. In all of those models, intra-organizational trust was the strongest mediator and due to its decrease, fake leadership negatively influences organization. Therefore, considering above literature review and those results, it should be assumed that intra-organizational trust will act as a mediator also in case of HEI. Hence, the following hypothesis can be proposed:

***H1b: The fake leadership is positively influencing organizational destruction through intra-organizational trust in HEIs.***

#### 1.2.4. The influence of fake leadership on organizational destruction through job performance in HEIs.

Many studies in the literature have explored both the positive and negative impacts of leadership on employees’ job performance. The are often assuming a direct correlation between individual performance and overall organizational outcomes, including the achievement of pre-set goals - an assumption well-established in management research [81]. Based on that, it is clear that factors negatively influencing job performance (such as fake leadership) will most probably translate into organizational destruction much easier through such diminished job performance. It may also be true for HEIs. However, job performance at HEIs is a complex issue [82, 83]. First and foremost, the combination of teaching and research activities in HEIs determines the dual nature of work at HEI. In addition, HEI employees are required to carry out (to a limited degree) work of an organizational and administrative nature, with the scope of this work determined by the range of functions performed at the HEI [82, 84]. The nature of each of these areas of work varies. Looking even at the classic job characteristics theory approach [85] significant differences can be found between them in relation to skill variety, task identity, task significance, autonomy and feedback from job.

Administrative (organizational) work is characterized by a high periodicity (identity) of tasks, and their diversity and autonomy depend on the level of management. Teaching work is also defined, with possible autonomy in content and techniques within the teaching areas. However, scientific work is always characterized by high autonomy, high diversity, low definition and usually also high importance [84].

These are special circumstances that require a favorable working environment, where providing security [86] and an appropriate organizational climate is the basis for unleashing creativity, innovation and unconventionality in solving scientific problems. Security and organizational climate should be considered in this case as a basic hygiene factor in the sense of Herzberg’s theory [87].

Fake leadership is adopting precisely defined actions against the employees and against the organization [12]. It has a destructive effect on the sense of security and destroys the favorable organizational climate. Hence, it makes not only scientific, but also didactic and organizational work much harder, especially since it negatively affects the results obtained by employees. There is also a mediating influence of organizational trust (which was discussed earlier), the destruction of which in the organization automatically contributes to an increase in the negative organizational climate and perceived job security, subsequently affecting the reduction of job performance and the destruction of the organization as a whole. Leaders’ lack of empathy, self-involvement, fragile self-esteem, and excessive sensitivity to criticism fosters an environment of distrust and fear. These traits manifest in behaviors such as creating and maintaining fear, humiliating employees, marginalizing or degrading them, exploiting their work, and destroying workplace relationships. Most critically, fake leaders engage in misleading and gaslighting employees, which also reduces intra-organizational trust, and further - undermines morale and reduces motivation. Negative outcomes of such behaviors for employees include “sense of being trapped, without any real means of either redressing or escaping the situation” [18]. The study of Azeez and Aboobaker [88] applied the Conservation of Resources (COR) theory. It indicates that toxic leadership behaviors (included in the concept of fake leadership) deplete employees from psychological resources such as self-esteem and confidence. This depletion can lead to demotivation and decreased job satisfaction, ultimately contributing to organizational destruction [49, 88]. All those job-related attitudes translate directly into decreasing job performance [89] – which is especially visible in HEIs [90, 91] – and it in turn increases organizational destruction, mainly due to lack of ability to link goals with actions. It remains in line with various models proposed by Bieńkowska and Tworek [12], who established that fake leadership influences job performance through the decrease of job-related attitudes and more importantly, increase of negative job-related attitudes.

Moreover, fake leadership contributes to organizational destruction because poor job performance further reduces productivity and employees in general are less able to contradict the destructive tendencies of authorities. Moreover, when they are subjected to negative behavior of a leader for doing “the right thing” and simultaneously constantly getting mixed messages about what is expected from them, they unvoluntary contribute to further organizational destruction.

Therefore, the following hypothesis can be proposed:

***H1: The fake leadership is positively influencing organizational destruction through intra-organizational trust and job performance in HEIs.***

## 2. Research methodology

The proposed hypotheses concerning fake leadership influence on organizational destruction through intra-organizational trust and job performance were verified through a structured, two-step empirical research process. The first step was aimed at assessing and refining the measurement tool – the survey, based on the panel of 25 competent judges. The second and main step was aimed at gathering data through corrected survey and verification of the hypotheses.

The main study was conducted in November 2024 among 500 employees of HEIs from Europe (France, Poland, Spain, and United Kingdom), based on mixed sources of data: purchased respondents panel and own network. The data were gathered using CAWI method (using SurveyMonkey) and respondents were asked to give informed, written consent. The acceptance of University Ethical Committee was obtained for the study (Ethical Committee of Wroclaw University of Science and Technology, decision no 24–26 (dated: 16/07/2024). Place of employment was the only variable limiting the sample – participants were asked a screening questions to confirm that they are active employees of HEIs. Respondents were employees on various positions (see Table 1) in various types of HEIs in each country (see Table 2). While the sample is not fully representative in a statistical sense, it is sufficiently large and diverse to allow for meaningful generalization within the context of European HEIs. Given that European universities operate within a common educational framework - shaped by policies such as the Bologna Process, shared accreditation standards, and similar governance structures - it is reasonable to generalize the findings within this regional context and it allows for the formulation of meaningful conclusions (see Table 1 and 2).

**Table 1:** Respondents overview.

		Gender			Total
		F	M	N/A	
**Position**	**Administrative staff**	51	73	2	126
	**Teaching-research staff**	98	164	1	263
	**Teaching staff**	38	68	7	113
	**Research staff**	8	16	3	27
**Total**		195	321	10	529

Source: own work

**Table 2:** Sample overview.

		Type of University		Total
		Public	Private	
**Country**	**France**	104	15	119
	**Poland**	64	7	71
	**Spain**	93	29	122
	**United Kingdom**	166	26	192
	**Lack of response**	23	2	25
**Total**		450	79	529

Source: own work

### 2.1. Variables overview

The following variables were included in the study in order to verify the proposed hypotheses:

**Fake Leadership** (FL) was measured on the 5-points’ Likert scale based on 20 items concerning each dimension of fake leadership (see S1 Table) based on Bieńkowska and Tworek (2024) measurement method.

**Intra-organizational trust** (OTrust) was measured on the 5-points’ Likert scale based on 5 items concerning various aspects of trust (between coworkers, between employees and supervisor and in self) (see S1 Table).

**Job Performance** (JPer) was measured on the 5-points’ Likert scale based on 4 items concerning various aspects of job performance (see S1 Table).

**Organizational destruction** (ODestr) was measured on the own 5-points’ Likert scale based on 8 items concerning each aspect of destruction (see S1 Table). As it is a new scale, never previously used, and developed specifically for HEI context, it underwent experts’ feedback at first, to make sure all relevant aspects of HEI functioning were taken into account. Next, it was verified using exploratory and confirmatory factor analysis, in order to include all relevant items, which account for a coherent construct.

To perform statistical analyses, all measurement scales employed in the study were thoroughly examined and validated. The process began with confirming that the collected data adhered to a normal distribution. Subsequently, the measurement scales were evaluated in IBM SPSS using three key statistical methods: Cronbach’s Alpha Analysis to assess the coherence and reliability of the measurement scales (value should exceed 0.7 [92]); Confirmatory Factor Analysis (CFA) to verify the internal consistency and coherence of the measurement scales (Average Variance Extracted (AVE), representing the proportion of variance explained by latent variables, was required to exceed 0.5); Kaiser-Meyer-Olkin (KMO) Test Analysis to evaluate the adequacy of the sample size for analysis using the measurement scales (values should exceed 0.5 [92]). Table 3 summarizes the results of these analyses. The results confirmed that the selected measurement scales demonstrated internal consistency, reliability, and coherence, making them suitable for further statistical procedures.

**Table 3:** Variables overview.

Variable name	Variable name	Number of items	AVE	K-M-O	Alpha Cronbach	N
Fake Leadership	FL	20	0,727	0,979	0,980	526
Intra-organizational trust	OTrust	5	0,783	0,860	0,931	524
Job Performance	JPer	4	0,852	0,856	0,942	528
Organizational Destruction	ODestr	8	0,758	0,954	0,955	520

Source: own work

Additionally, discriminant validity was assessed to ensure that the latent variables, representing distinct theoretical constructs, were statistically different. The Heterotrait-Monotrait Ratio (HTMT) values were below 0.65 [93]. This result confirms the appropriateness of the variables for subsequent analyses, such as correlation, regression, and path analysis. Moreover, common method bias, which occurs when data for both independent and dependent variables are collected from the same source and under the same measurement conditions, was assessed with Harman’s Single-Factor Test [94]. The analysis revealed that the single factor accounted for 22.7% of the variance, well below the 50% threshold. This result confirms that common method bias is not present in the dataset.

## 3. Research results

### 3.1. Regression and correlation analysis

The statistical analysis in the process of hypotheses verification started with developing a linear regression model calculated in IBM SPSS (based on S2 File) to: (1) determine whether the independent variable, fake leadership, has a statistically significant impact on organizational destruction while controlling for other factors (organizational characteristics from Leavitt’s model) and (2) verify the lack of presence of multicollinearity among the variables within the sample using the Variance Inflation Factor (VIF) (values should remain below 5.0 to indicate no multicollinearity issue [95]).

The obtained model was statistically significant, characterized by corrected R2 = 0,713 and F(7,499) = 180,395, p <0,001. The statistics concerning each variable in regression model are given in Table 4. The obtained values of VIF confirm that there is no multicollinearity issue. Moreover, the regression analysis shows that fake leadership is statistically significant independent variable in the model obtained for organizational destruction, while controlling for environment uncertainty, structure complexity, culture strength and services diversity within the given HEI (CV1-CV4).

**Table 4:** Regression model overview.

Model	Non-standardized coefficients		Standardized	t	p	VIF
	B	s.e.	Beta			(MC)
()	3.689	0.221		16.657	<0.001	
FL	0.298	0.031	0.312	9.470	<0.001	2.803
OTrust	–0.323	0.036	–0.342	–9.082	<0.001	3.661
JPer	–0.240	0.031	–0.266	–7.648	<0.001	3.107
CV1	0.128	0.026	0.112	4.990	<0.001	1.290
CV2	0.002	0.028	0.002	0.081	0.936	1.397
CV3	–0.048	0.030	–0.040	–1.623	0.105	1.531
CV4	0.006	0.028	0.005	0.223	0.823	1.443

Next, in order to match the requirements for mediation analysis, the r-Pearson correlation analysis was performed, and its results are given in table 5. The results clearly show that predictor (FL), mediators (intra-organizational trust and job performance) and outcome variable (organizational destruction) are all correlated, which allows to carry on with mediation analysis.

**Table 5:** Correlation analysis.

		FL	OTrust	JPer	ODestr
**FL**	r-Perason	1	–0.777	–0.737	0.807
	p		<0.001	<0.001	<0.001
	N	522	522	521	516
**OTrust**	r-Perason	–0.777	1	0.793	–0.842
	p	<0.001		<0.001	<0.001
	N	522	530	529	524
**JPer**	r-Perason	–0.737	0.793^**^	1	–0.803
	p	<0.001	<0.001		<0.001
	N	521	529	529	523
**ODestr**	r-Perason	0.807	–0.842^**^	–0.803	1
	p	<0.001	<0.001	<0.001	
	N	516	524	523	524

### 3.2. Mediation analysis

Those results allowed for the development of a mediation model calculated in IBM SPSS Macro Process (model 6). As a first step, the regression model for intra-organizational trust as an outcome variable was calculated, with FL as a predictor (table 6 showing the model overview and statistics and table 7 showing regression model coefficients). The model was statistically significant and well-fitted (see table 6), with corrected R2 = 0,6052. FL was a statistically significant predictor of intra-organizational trust (see table 7) with coeff. = -0,7840, p < 0,001, which states what FL significantly, negatively influences intra-organizational trust within the mediation model.

**Table 6:** Regression model overview (intra-organizational trust).

Model Summary	Value
R	0.7780
R-sq	0.6052
MSE	0.5657
F	786.4898
df1	1.0000
df2	513.0000
p	< 0.001

**Table 7:** Regression model coefficients (intra-organizational trust).

Model	Coeff	SE	t	p	LLCI	ULCI
constant	5.1527	0.0904	57.0159	< 0.001	4.9752	5.3303
FL	–0.7840	0.0280	–28.0444	< 0.001	–0.8390	–0.7291

As a second step, the regression model for job performance as an outcome variable was calculated, with FL and intra-organizational trust as predictors (table 8 showing the model overview and statistics and table 9 showing regression model coefficients). The model was statistically significant and well-fitted (see table 8), with corrected R2 = 0,6682. FL was a statistically significant predictor of job performance (see table 9) with coeff. = -0,3112, p < 0,001, which states what FL significantly, negatively influences job performance within the mediation model. Intra-organizational trust was a statistically significant predictor of job performance (see table 9) with coeff. = 0,5930, p < 0,001, which states what intra-organizational trust significantly, positively influences job performance within the mediation model. Therefore, those results allow to accept hypotheses H1a, which state that ***fake leadership is positively influencing organizational destruction in HEIs.***

**Table 8:** Regression model overview (job performance).

Model Summary	Value
R	0.8174
R-sq	0.6682
MSE	0.5218
F	515.4772
df1	2.0000
df2	512.0000
p	< 0.001

**Table 9:** Regression model coefficients (job performance).

Model	Coeff	SE	t	p	LLCI	ULCI
constant	2.2182	0.2351	9.4350	< 0.001	1.7563	2.6801
FL	–0.3112	0.0427	–7.2809	< 0.001	–0.3951	–0.2272
OTrust	0.5930	0.0424	13.9842	< 0.001	0.5097	0.6763

As a third step, the regression model for organizational destruction as an outcome variable was calculated, with FL, intra-organizational trust and job performance as predictors (table 10 showing the model overview and statistics and table 11 showing regression model coefficients). The model was statistically significant and well-fitted (see table 10), with corrected R2 = 0,7926. FL was a statistically significant predictor of organizational destruction (see table 11) with coeff. = 0,2933, p < 0,001, which states what FL significantly, positively influences organizational destruction within the mediation model. Intra-organizational trust was a statistically significant predictor of organizational destruction (see table 11) with coeff. = -,03673, p < 0,001, which states what intra-organizational trust significantly, negatively influences organizational destruction within the mediation model. Job performance was a statistically significant predictor of organizational destruction (see table 11) with coeff. = -,02424, p < 0,001, which states what intra-organizational trust also significantly, negatively influences organizational destruction within the mediation model. Therefore, those results allow to accept hypotheses H1b, which state that ***fake leadership is positively influencing organizational destruction through intra-organizational trust in HEIs.***

**Table 10:** Regression model overview (organizational destruction).

Model Summary	Value
R	0.8903
R-sq	0.7926
MSE	0.2662
F	651.1051
df1	3.0000
df2	511.0000
p	< 0.001

**Table 11:** Regression model coefficients (organizational destruction).

Model	Coeff	SE	t	p	LLCI	ULCI
constant	4.0950	0.1819	22.5078	< 0.001	3.7376	4.4524
FL	0.2933	0.0321	9.1460	< 0.001	0.2303	0.3563
OTrust	-0.3674	0.0356	-10.3192	< 0.001	-0.4374	-0.2975
JPer	-0.2424	0.0316	-7.6779	< 0.001	-0.3044	-0.1803

Therefore, the obtained results confirmed that all assumed relations within the mediation model were statistically significant. Table 12 shows direct and indirect effects occurring within the final model, showing that all assumes paths were statistically significant (both BootLLCI and BootULCI are above 0) and both intra-organizational trust and job performance are statistically significant mediators of the relation between FL and organizational destruction.

**Table 12:** Direct and Indirect Effects within the model.

Effect Type	Effect	BootSE	BootLLCI	BootULCI
Total Direct	0.2933	0.0321	0.2303	0.3563
Total Indirect	0.4762	0.0339	0.4091	0.5419
Ind1 (FL → OTrust → ODestr)	0.2881	0.0340	0.2216	0.3558
Ind2 (FL → JPer → ODestr)	0.0754	0.0161	0.0473	0.1094
Ind3 (FL → OTrust → JPer → ODestr)	0.1127	0.0182	0.0788	0.1499

Therefore, the findings support the acceptance of Hypothesis H1, which state that ***fake leadership is positively influencing organizational destruction through intra-organizational trust and job performance in HEIs.*** It should be noted that the cross-sectional design of the study has some limitations in fully establishing a causality between fake leadership and organizational destruction. However, empirical study was coupled with a critical literature review as a basis for hypotheses development and together it is enough to establish the causality.

## 4. Discussion

**Fig. 1:**
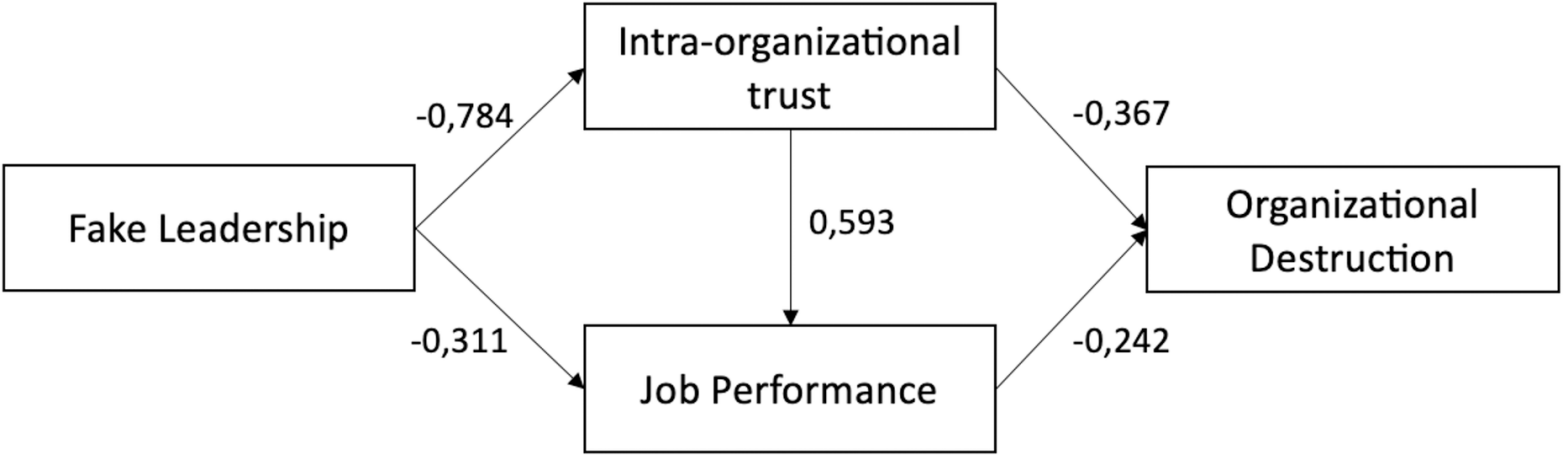
The model of fake leadership influence on organizational destruction in HEIs.

The study addressed a research gap in the literature, allowing to include fake leadership as one of destructive leadership styles existing in HEIs, by exploring the relation between fake leadership and organizational destruction in HEIs. While prior research has extensively examined the positive impacts of leadership styles on HEI performance (e.g., [16, 17]), studies on the negative effects of destructive leadership styles are significantly limited, particularly within HEIs, and there is no systemic approach allowing to analyze destructive leadership styles or propose a typology or a matrix of specific destructive leadership styles. Therefore, building on prior works (e.g., [12]), this study contributes a novel perspective by introducing fake leadership as a distinct construct within the spectrum of destructive leadership styles in HEIs, emphasizing the intent to hide negative behaviors while projecting an image of authenticity. Moreover, it extends the work of Einarsen [46], Schyns and Shilling [14] and Mackey et al. [45] on destructive leadership styles by proposing and empirically verifying not only a construct but a model specific for HEIs that links a specific type of destructive leadership style - fake leadership - to organizational destruction through the mediating effects of intra-organizational trust and job performance (due to the negative effects of fake leadership on both of them) (see **Fig. 1**).

The study also contributes to the further development of The Toxic Triangle Framework [23, 69], which explores the interplay between leader traits, follower susceptibility, and conducive organizational environments, which served as a foundation for understanding how HEIs are uniquely vulnerable to fake leadership. The obtained results confirm that and extend this framework by demonstrating how fake leadership exploits these dynamics, specifically through its dual impact on intra-organizational trust and job performance, to foster not only negative effect on organization outcomes, but organizational destruction (understood in a way tailored to the HEIs specificity).

Therefore, this research makes several important contributions to the theory and literature on leadership and HEI organizational studies: (1) The concept of fake leadership was confirmed to be one of the destructive leadership styles in HEIs with its highlighted role of hidden intent and the duality of a leader’s behavior - presenting as authentic while engaging in destructive practices. This nuanced perspective enriches leadership theories by bridging the gap between overt and covert destructive behaviors in HEIs, confirming that those covert ones actively cause organizational destruction. (2) The study proposes the notion of organizational destruction tailored specifically for HEIs, providing empirical evidence linking fake leadership to systemic inefficiencies, misaligned goals, and loss of trust—issues that have been discussed anecdotally in prior studies (e.g., 21, 22]) and showing that it creates a clear mechanism of destruction enabled by fake leadership in organization. (3) The findings underscore the critical role of intra-organizational trust and job performance as mediators in the relation between fake leadership and organizational destruction. It shows that fake leadership negatively influences intra-organizational trust and job performance and it has further negative consequences for the organization, causing its destruction. This offers a deeper understanding of the mechanisms through which leadership behaviors negatively influence organizational outcomes. (4) By situating the discussion within HEIs, the study highlights the unique vulnerabilities of these institutions to fake leadership (previously suggested by Ryan et al. [18] or Dopson [48]), particularly their reliance on academic ethos, collegiality, and trust. It emphasizes the need for leadership models that align with the values and missions of HEIs.

The findings also have significant implications for HEIs, which hold a unique responsibility to uphold ethical leadership as institutions dedicated to truth and societal progress. It confirms that HEIs are susceptible for destructive leadership styles, especially fake leadership (which was proven to be a style, which causes organizational destruction in HEIs), due to hierarchical structures and resource constraints and possibility of such pathology should be addressed. Promoting and ensuring authentic leadership should be a priority, focusing on fostering trust, collaboration, and thriving academic environments. Rigorous leadership selection processes must evaluate ethical alignment, and training programs should emphasize transparency and accountability. Finally, it shows that combating fake leadership requires proactive measures, such as whistleblowing mechanisms, leadership audits, and organizational climate assessments, to safeguard institutional integrity and performance.

## Conclusions

The aim of this paper was to **examine the role of fake leadership in creating organizational destruction of HEIs. Such aim was fulfilled, which allowed to narrow the indicated research gap and include fake leadership into the group of destructive leadership styles occurring in HEIs by** exploring and confirming the impact of fake leadership on organizational destruction in HEIs through its negative effect on intra-organizational trust and job performance. The study extends existing theories, such as the Toxic Triangle Framework [23, 69], by demonstrating how fake leadership exploits trust deficits and undermined job performance, ultimately fostering organizational destruction in the specific organizational environment. This research also contributes to the literature by providing empirical evidence on how destructive leadership styles (i.e., fake leadership) undermine HEIs integrity, systemic coherence, and operational efficiency, fostering organizational destruction, understood in a way tailored for HEIs.

The study also offers profound implications for HEIs. As institutions committed to truth and societal progress, HEIs must prioritize combatting fake leadership to safeguard their missions. By addressing fake leadership, HEIs can reinforce trust, promote collaboration, and secure their long-term sustainability in an increasingly competitive and resource-constrained environment, preventing organizational destruction.

This study has several limitations that should be acknowledged. Its geographical scope focuses on HEIs in Europe, limiting the generalizability of the findings to institutions in other cultural and organizational contexts. The sample, while diverse, is not fully representative. Furthermore, the research examines only two mediators—intra-organizational trust and job performance—leaving other potential factors, such as governance structures or external pressures, unexplored.

Future research should address these limitations by expanding to diverse global regions, employing longitudinal designs to explore long-term impacts. Future studies should also explore interventions to mitigate fake leadership. However, mainly, this study serves as a foundation for future research on leadership pathologies in HEIs and underscores the importance of ethical leadership for organizational resilience and success.

## Supporting information

S1 Table Questionnaire items. (DOCX)

S2 File Data set for the analysis. (XLSX)

## References

[1] Gallos JV, Bolman LG. Reframing academic leadership. John wiley & sons. 2021.

[2] Srivastava AP, Mani V, Yadav M, Joshi Y. Authentic leadership towards sustainability in higher education–an integrated green model. International Journal of Manpower. 2020;41(7):901–23.

[3] Fraser S. Authentic leadership in higher education: Influencing the development of future leaders. Journal of Leadership Studies. 2014;10(2):123–45. doi: 10.1002/jls.21345

[4] Bolden R, Petrov G, Gosling J. Distributed leadership in higher education: Rhetoric and reality. Educational Management Administration & Leadership. 2009;37(2):257–77. doi: 10.1177/1741143208100301

[5] Henkel M. Emerging concepts of academic leadership and their implications for intra‐institutional roles and relationships in higher education. Euro J of Education. 2002;37(1):29–41. doi: 10.1111/1467-3435.00089

[6] Shulman S. Contingency in higher education: Evidence and explanation. Academic Labor: Research and Artistry. 2017;1(1):3.

[7] Loo M. Academic leadership and governance of higher education: A guide for trustees, leaders, and aspiring leaders of two-and four-year institutions. Canadian Journal of University Continuing Education. 2014;40(2).

[8] Hart PF, Rodgers W. Competition, competitiveness, and competitive advantage in higher education institutions: a systematic literature review. Studies in Higher Education. 2024;49(11):2153–77. doi: 10.1080/03075079.2023.2293926

[9] Taylor J. The state and higher education institutions: New pressures, new relationships and new tensions. Higher education and the state: Changing relationships in Europe and East Asia. 2013:9–35.

[10] Alvesson M, Kärreman D. Uncreative destruction: Competition and positional games in higher education. In The corporatization of the business school. Routledge. 2017:111–27.

[11] Karger H. How ego, greed, and hubris (almost) destroyed a university: Implications for academic freedom. AAUP Journal of Academic Freedom. 2020;11.

[12] Bieńkowska A, Tworek K. (2024). Leadership styles and job performance: the impact of fake leadership on organizational reliability, New York: Routledge. 10.4324/9781032664194

[13] Einarsen S, Aasland M. S, & Skogstad A. (2016). The nature and outcomes of destructive leadership behavior in organizations. In Risky business (pp. 323-349). Routledge.

[14] Schyns B, Schilling J. How bad are the effects of bad leaders? A meta-analysis of destructive leadership and its outcomes. The Leadership Quarterly. 2013;24(1):138–58.

[15] Siddique A, Aslam HD, Khan M, Fatima U. Impact of academic leadership on faculty’s motivation and organizationaleffectiveness in higher education system. International journal of academic research. 2011;3(3). doi: DOIORIDENTIFIER

[16] Alonderiene R, Majauskaite M. Leadership style and job satisfaction in higher education institutions. International Journal of Educational Management. 2016;30(1):140–64.

[17] Iqbal Q, & Piwowar-Sulej K. (2022). Sustainable leadership in higher education institutions: social innovation as a mechanism. International Journal of Sustainability in Higher Education, 23(8), 1-20.

[18] Ryan P, Odhiambo G, Wilson R. Destructive leadership in education: A transdisciplinary critical analysis of contemporary literature. International Journal of Leadership in Education. 2021;24(1):57–83.

[19] Smith N, Fredricks-Lowman I. Conflict in the workplace: A 10-year review of toxic leadership in higher education. International Journal of Leadership in Education. 2020;23(5):538–51.

[20] Balwant PT. The dark side of teaching: destructive instructor leadership and its association with students’ affect, behaviour, and cognition. International Journal of Leadership in Education. 2017;20(5):577–604.

[21] Klaus K, Steele SL. An exploratory and descriptive study of destructive leadership in US higher education. International Journal of Leadership in Education. 2022;25(5):704–24.

[22] Ghamrawi N, Abu-Shawish RK, Shal T, Ghamrawi NA. Destructive leadership behaviors: The case of academic middle leaders in higher education. International Journal of Educational Research. 2024;126:102382. doi: 10.1016/j.ijer.2024.102382

[23] Thoroughgood CN, Padilla A. Destructive leadership and the Penn State scandal: A toxic triangle perspective. Industrial and Organizational Psychology. 2013;6(2):144–9.

[24] Elia G, Secundo G, Passiante G. Pathways towards the entrepreneurial university for creating entrepreneurial engineers. An Italian case. International Journal of Entrepreneurship and Innovation Management. 2017;21(1/2):27–48. doi: 10.1504/IJEIM.2017

[25] Brzeziński J. Etos akademicki – między tradycją i wyzwaniami współczesności. Gdańsk, 2010.

[26] Bieńkowska. Dynamic perspective of academic values of modern university. W: Dynamic capabilities and their strategic dimension: aspects of imitation and innovation [in:] (eds.) Krzakiewicz K, Cyfert Sz. Poznań University of Economics and Business Press, Poznań, 2019. p. 9–22.

[27] Hildesheim C, Sonntag K. The Quality Culture Inventory: a comprehensive approach towards measuring quality culture in higher education. Studies in Higher Education. 2019;45(4):892–908. doi: 10.1080/03075079.2019.1672639

[28] Agasisti T. Management of higher education institutions and the evaluation of their efficiency and performance. Tertiary Education and Management. 2017;23(3):187–90. doi: 10.1080/13583883.2017.1336250

[29] Breakwell GM, Tytherleigh MY. University leaders and university performance in the United Kingdom: is it ‘who’ leads, or ‘where’ they lead that matters most?. High Educ. 2010;60(5):491–506. doi: 10.1007/s10734-010-9311-0

[30] Peus C, Welpe I, Weisweiler S, Frey D. Führung an Hochschulen. In Felfe J. (Ed.), Trends der psychologischen Führungsforschung: Neue Konzepte, Methoden und Erkenntnisse, Psychologie für das Personalmanagement. Hogrefe. 2015, pp. 527–39.

[31] Ugwu CI, Okore AM. Transformational and transactional leadership influence on knowledge management activities of librarians in university libraries in Nigeria. Journal of Librarianship and Information Science. 2020;52(3):864–79. doi: 10.1177/0961000619880229

[32] Ambad SNA, Kalimin KM, Damit DHDA, Andrew JV. The mediating effect of psychological empowerment on leadership styles and task performance of academic staff. Leadership & Organization Development Journal. 2021;42(5):763–82. doi: 10.1108/LODJ-05-2020-0197

[33] Carvalho SW, de Oliveira Mota M. The role of trust in creating value and student loyalty in relational exchanges between higher education institutions and their students. Journal of Marketing for Higher Education. 2010;20(1):145–65. doi: 10.1080/08841241003788201

[34] Herbst THH, Roux T. Toxic leadership: a slow poison killing women leaders in higher education in South Africa?. High Educ Policy. 2021;36(1):164–89. doi: 10.1057/s41307-021-00250-0

[35] Brewer GA, Selden SC. Why elephants gallop: Assessing and predicting organizational performance in federal agencies. Journal of public administration research and theory. 2000;10(4):685–712.

[36] Rowley DJ, Sherman H. The special challenges of academic leadership. Management Decision. 2003;41(10):1058–63. doi: 10.1108/00251740310509580

[37] Nasrun DFPA, Nasution NB, Tambunan HP. The effect of dean leadership, on the performance of lecturers working in the faculty of education in Medan State University. International Journal of Innovation, Creativity and Change. 2019;5(5):515–30.

[38] Saad MA. The relationship between strategic leadership and intellectual capital management: Evidence from the faculty members at the Northern Border University. Int J Adv Appl Sci. 2020;7(5):27–38. doi: 10.21833/ijaas.2020.05.005

[39] Rehman U, Iqbal A. Nexus of knowledge-oriented leadership, knowledge management, innovation and organizational performance in higher education. Business Process Management Journal. 2020;26(6):1731–58. doi: 10.1108/BPMJ-07-2019-0274

[40] Angelo R, McCarthy R. A pedagogy to develop effective virtual teams. Journal of Computer Information Systems. 2020;61(5):450–7. doi: 10.1080/08874417.2020.1717396

[41] Meslec N, Curseu PL, Fodor OC, Kenda R. Effects of charismatic leadership and rewards on individual performance. The Leadership Quarterly. 2020;31(6):101423. doi: 10.1016/j.leaqua.2020.101423

[42] Bianchi C, Nasi G, Rivenbark WC. Implementing collaborative governance: models, experiences, and challenges. Public Management Review. 2021;23(11):1581–9.

[43] Leal Filho W, Eustachio JHPP, Caldana ACF, Will M, Lange Salvia A, Rampasso IS, et al. Sustainability leadership in higher education institutions: An overview of challenges. Sustainability. 2020;12(9):3761.

[44] Harris A, Jones M, Ismail N. Distributed leadership: taking a retrospective and contemporary view of the evidence base. School Leadership & Management. 2022;42(5):438–56. doi: 10.1080/13632434.2022.2109620

[45] Mackey JD, Parker Ellen B III, McAllister CP, Alexander KC. The dark side of leadership: A systematic literature review and meta-analysis of destructive leadership research. Journal of Business Research. 2021;132:705–18. doi: 10.1016/j.jbusres.2020.10.037

[46] Einarsen S, Aasland MS, Skogstad A. Destructive leadership behaviour: A definition and conceptual model. The Leadership Quarterly. 2007;18(3):207–16. doi: 10.1016/j.leaqua.2007.03.002

[47] Başkan B. Toxic leadership in education. International Journal of Educational Administration, Management, and Leadership. 2020:97–104.

[48] Dopson S, Ferlie E, McGivern G, Fischer MD, Mitra M, Ledger J, et al. Leadership development in Higher Education: A literature review and implications for programme redesign. Higher Education Quarterly. 2018;73(2):218–34. doi: 10.1111/hequ.12194

[49] Erickson A, Shaw B, Murray J, Branch S. Destructive leadership: Causes, consequences and countermeasures. Organizational Dynamics. 2015;44(4):266–72. doi: 10.1016/j.orgdyn.2015.09.003

[50] Krasikova DV, Green SG, LeBreton JM. Destructive leadership: A theoretical review, integration, and future research agenda. Journal of Management. 2013;39(5):1308–38. doi: 10.1177/0149206312471388

[51] Mouzelis NP. Organizational pathology: Life and death of organizations. Routledge. 2017.

[52] Habibi Doost M, Fadavi M, Farhadi H. Identification and pathology of organizational syndromes in education. Iranian journal of educational sociology. 2021;4(2):127–38.

[53] Rafiei S, Nejatifar Z, Alizadeh A, Amerzadeh M, Babajamadi S, Farmani M, et al. The relationship between organizational pathology and employees’ organizational commitment in Qazvin University of Medical Sciences. Journal of Health Reports and Technology. 2022;8(4).

[54] Özdemir TY, Yalçın AY. Organizational pathologies in educational organizations and administrator responses to pathologies. Journal of Faculty of Educational Sciences. 2023;56(3).

[55] Staw BM, McKechnie PI, Puffer SM. The justification of organizational performance. Administrative science quarterly, 1983. 582-600.

[56] Ahmed A, Khuwaja FM, Brohi NA, Othman I, Bin L. Organizational factors and organizational performance: A resource-based view and social exchange theory viewpoint. International Journal of Academic Research in Business and Social Sciences, 2018;8(3):579–599.

[57] Bieńkowska A, Tworek K, Zabłocka-Kluczka A. Organizational reliability model verification in the crisis escalation phase caused by the COVID-19 pandemic. Sustainability. 2020;12(10):4318.

[58] Lester DL, Parnell JA, Carraher S. Organizational life cycle: A five‐stage empirical scale. The international journal of organizational analysis. 2003;11(4):339–54.

[59] Schilling J. From ineffectiveness to destruction: A qualitative study on the meaning of negative leadership. Leadership. 2009;5(1):102–28.

[60] Richins ML. A multivariate analysis of responses to dissatisfaction. Journal of the academy of marketing science. 1987;15(3):24–31.

[61] Falout J, Elwood J, Hood M. Demotivation: Affective states and learning outcomes. System. 2009;37(3):403–17.

[62] Leavitt HJ, Whisler TL. Management in the 1980’s. Harvard Business Review. 1958;36:41–8.

[63] Paul DA. Higher education in competitive markets: Literature on organizational decline and turnaround. The Journal of General Education. 2005;54(2):106–38.

[64] Meyer JW, Ramirez FO, Frank DJ, Schofer E. Higher education as an institution. Sociology of higher education: Contributions and their contexts, 2007. 187.

[65] Croxford L, Raffe D. The iron law of hierarchy? Institutional differentiation in UK higher education. Studies in Higher Education. 2014;40(9):1625–40. doi: 10.1080/03075079.2014.899342

[66] Pusser B. Power and authority in the creation of a public sphere through higher education. In Universities and the public sphere. Routledge. 2012:pp. 27–46.

[67] Bowen WG, Tobin EM. Locus of authority: The evolution of faculty roles in the governance of higher education. Princeton University Press.2015.

[68] Marini G, Videira P, Carvalho T. Is new public management redefining professional boundaries and changing power relations within higher education institutions?. Journal of the European Higher Education Area. 2016;2016(3).

[69] Padilla A, Hogan R, Kaiser RB. The toxic triangle: Destructive leaders, susceptible followers, and conducive environments. The Leadership Quarterly. 2007;18(3):176–94. doi: 10.1016/j.leaqua.2007.03.001

[70] Schneider CS. The toxic triangle: A qualitative study of destructive leadership in public higher education institutions. 2021.

[71] Li PP. Towards an interdisciplinary conceptualization of trust: a typological approach. Manag Organ Rev. 2007;3(3):421–45. doi: 10.1111/j.1740-8784.2007.00081.x

[72] Mayer RC, Gavin MB. Trust in management and performance: who minds the shop while the employees watch the boss?. AMJ. 2005;48(5):874–88. doi: 10.5465/amj.2005.18803928

[73] Bieńkowska A, Walecka-Jankowska K, Zabłocka-Kluczka A, Zimmer J. Influence of intraorganizational trust on organizational outcomes. Operations Research and Decisions. 2018;28.

[74] Tierney WG. (2008). Trust and organizational culture in higher education. In Cultural perspectives on higher education (pp. 27-41). Dordrecht: Springer Netherlands.

[75] Vidovich L, Currie J. Governance and trust in higher education. Studies in Higher Education. 2011;36(1):43–56.

[76] Jameson J, Barnard J, Rumyantseva N, Essex R, Gkinopoulos T. A systematic scoping review and textual narrative synthesis of trust amongst staff in higher education settings. Studies in Higher Education. 2023;48(3):424–44.

[77] Tapper T, Palfreyman D. Understanding collegiality: the changing Oxbridge model. Tertiary Education & Management. 2002;8(1):47–63.

[78] Serpa S, Sá MJ. Trust in Higher Education Management and Organizational Culture. JESR. 2022;12(1):8. doi: 10.36941/jesr-2022-0002

[79] Sahoo R, Sahoo CK. Organizational justice, conflict management and employee relations. IJM. 2019;40(4):783–99. doi: 10.1108/ijm-12-2017-0342

[80] Maassen P, Stensaker B (2022). Trust and higher education governance in Norway and the United Kingdom. In Trusting in Higher Education: A multifaceted discussion of trust in and for higher education in Norway and the United Kingdom (pp. 17-36). Cham: Springer International Publishing.

[81] Schaufeli WB. Engaging leadership in the job demands-resources model. Career Development International. 2015;20(5):446–63. doi: 10.1108/cdi-02-2015-0025

[82] Edwards JA, Van Laar D, Easton S, Kinman G. The work‐related quality of life scale for higher education employees. Quality in Higher Education. 2009;15(3):207–19. doi: 10.1080/13538320903343057

[83] Nazir O, Islam JU. Enhancing organizational commitment and employee performance through employee engagement. SAJBS. 2017;6(1):98–114. doi: 10.1108/sajbs-04-2016-0036

[84] Chen S, Yang C, Shiau J, Wang H. The development of an employee satisfaction model for higher education. The TQM Magazine. 2006;18(5):484–500. doi: 10.1108/09544780610685467

[85] Hackman JR, Oldham GR. Development of the job diagnostic survey. Journal of Applied Psychology. 1975;60(2):159.

[86] Hackman R, Lawler E, Oldham G. Job characteristics theory. Organizational Behavior. 2015;1:75–93.

[87] Malik ME, Naeem B. Towards understanding controversy on Herzberg theory of motivation. World Applied Sciences Journal. 2013;24(8):1031–6.

[88] Azeez F, Aboobaker N. Echoes of dysfunction: a thematic exploration of toxic leadership in higher education. Journal of Aggression, Conflict and Peace Research. 2024;16(4):439–56.

[89] Diamantidis AD, Chatzoglou P. Factors affecting employee performance: an empirical approach. International journal of productivity and performance management. 2018;68(1):171–93.

[90] Deligero JCL, Laguador JM. Work engagement among employees and its relationship with work units’ performance of a higher education institution. International Journal of Management Sciences. 2014;3(12):909–17.

[91] Tashliyev A, Tirtoprojo S. Examining the factors affecting employee performance of higher education institution employee in the new normal era. International Journal of Economics and Business Issues. 2023;2(1):47–57.

[92] Drost EA. Validity and reliability in social science research. Education Research and Perspectives. 2011;38(1):105–23.

[93] Ab Hamid MR, Sami W, Sidek MM. Discriminant validity assessment: Use of Fornell & Larcker criterion versus HTMT criterion. Journal of Physics: Conference Series. 2017;890(1):012163.

[94] Aguirre-Urreta MI, Hu J. Detecting common method bias: Performance of the Harman’s single-factor test. ACM SIGMIS database: the DATABASE for Advances in Information Systems. 2019;50(2):45–70.

[95] Midi H, Bagheri A (2010). Robust multicollinearity diagnostic measure in collinear data set. In Proceedings of the 4th international conference on applied mathematics, simulation, modeling (pp. 138–142). Stevens Point, WI: World Scientific and Engineering Academy and Society (WSEAS).

