# Supplementary material for: Fake leadership influence on organizational destruction in Higher Education Institutions (HEIs)
Source: PLoS One. 2025 Apr 23;20(4):e0321194. doi: 10.1371/journal.pone.0321194 (PMC12017485; doi:10.1371/journal.pone.0321194)
Supplement: S1 Table — (DOCX) [file pone.0321194.s001.docx]

**Table 1. Questionnaire Items.** Source: own work

| **Statement items used for Fake Leadership assessment (on a 5’points Likert scale):**   - Lack of empathy coupled with lack of the ability to feel guilt or remorse towards others - Self-involvement (self-centeredness) seen as prioritizing own goals and needs, fulfilling them with disregard to others (employees, environment) - Fragile high self-esteem coupled with all sorts of insecurities - Over-sensitivity to criticism and need of approval causing the need to surrounding oneself by flatterers and yes-sayers - Intention to win coupled with the low sense of responsibility including shifting responsibility and seeking someone else to blame for own mistakes - Insincerity (demonstrates beliefs that are not consistent with actions) and the ability to pretend - Inducing and sustaining fear among employees by e.g. threats, verbal abuse, intimidation, punitive feedback - Humiliating employees by e.g. public criticism, mockery, insults, withholding praise, setting unreasonable expectations - Gathering information to use against employees by e.g. surveillance, personal interactions, monitoring communication - Demeaning, marginalizing, or degrading employees - Blocking employees and their initiatives, especially those employees seen as competition - Exploiting employees by withholding the credit to the employees, when they deserve it, taking the credit for the employees’ work and blaming own mistakes or lack of performance on employees - Destruction of employees’ relations by e.g. pitting them agains each other, spreading rumors, rewarding conflict - Misleading and gaslighting employees by e.g. denying, minimizing or belittling their views and creating confusion (including blaming the victims) - Manipulation of information by e.g. sharing untrue information, selectively given information or withholding important information - Lack of communication and transparency by e.g. limiting access to information, avoiding documentation, using ambiguous language - Limiting access to information - Lack of transparency of decisions and regarding the operation of the University and employees - Fickleness and lack of consistency e.g. sudden and unsubstantiated changes in decisions (also made by others), arbitrary rules, sudden changes in priorities, lack of follow-through of decisions - Communities’ destruction by e.g. isolating, scapegoating, undermining shared values, intimidating |
| --- |
| **Statement items used for Intra-organizational trust assessment (on a 5’points Likert scale):**   - I am treated fairly by my University. - I trust management to look after my best interests. - I trust myself. - I consider myself as trustworthy. - I trust my coworkers. |
| **Statement items used for Job Performance assessment (on a 5’points Likert scale):**   - My job quality is: - My job efficiency is: - My punctuality is: - My effectiveness of achieving the set goals is: |
| **Statement items used for Organizational destruction assessment (on a 5’points Likert scale):**   - The goals of the University at all levels are not linked to real actions. - I feel that University Authorities (Rector, Vice-Rectors, Deans and other members of authorities) are striving for the destruction of the organization. - I feel that University Authorities (Rector, Vice-Rectors, Deans and other members of authorities) act randomly. - I feel that University Authorities (Rector, Vice-Rectors, Deans and other members of authorities) act chaotically. - I have reservations about the managerial competence of University Authorities (Rector, Vice-Rectors, Deans and other members of authorities). - Decisions at the University are made in isolation from formal organizational structures and formal processes. - Changes in the structure are, in my opinion, chaotic and irrational. - Technological solutions used at my University are outdated. |
